# An Investigation on the Impact of Unloading Rate on Coal Mechanical Properties and Energy Evolution Law

**DOI:** 10.3390/ijerph19084546

**Published:** 2022-04-09

**Authors:** Hongjun Guo, Zhongguang Sun, Ming Ji, Yongfeng Wu, Lihui Nian

**Affiliations:** 1 Jiangsu Vocational Institute of Architectural Technology, Xuzhou 221116, China; hongjun_g@163.com (H.G.); wyf_617@126.com (Y.W.); nlh18260715645@126.com (L.N.); 2 State Key Laboratory of The Gas Disaster Detecting, Preventing and Emergency Controlling, Chongqing 400037, China; sunzhongguang126@126.com; 3 China Coal Technology and Engineering Group Chongqing Research Institute, Chongqing 400039, China; 4 Key Laboratory of Deep Coal Resource Mining, Ministry of Education of China, School of Mines, China University of Mining & Technology, Xuzhou 221116, China

**Keywords:** coal body, the loading and unloading confining pressure of the shaft, the unloading rate of the confining pressure, mechanical properties, energy characteristics, roadway excavation

## Abstract

In order to further explore the relationship between the excavation speed and the damage of surrounding rocks and dynamic manifestation, the stress paths of unloading confining pressure and loading axial pressure were designed based on the changes in the roadway surrounding rock stress in this study. Additionally, the mechanical properties and energy evolution law of the coal body were investigated under various unloading rates. As the unloading rate increased, the mechanical properties of the coal body including the failure strength, the confining pressure, the axial strain, and horizontal strain tended to decrease at the rupture stage, while the volume strain and the elastic modulus increased, indicating that the rupture form evolved from the ductile failure to brittle failure. Regarding the energy, the axial pressure did positive work while the confining pressure did negative work, with the total work and the stored elastic strain energy decreasing. In addition, with the increase of the dissipation energy, the elastic strain energy conversion rate decreased linearly, indicating that the high unloading rate increased the possibility of dynamic disasters induced by the instantaneous brittle rupture of the coal body. On the other hand, due to the low releasable elastic strain energy stored in the coal body, the strength and probability of subsequent dynamic manifestation of coal body destruction were reduced. Therefore, increasing the excavation speed in a controllable way can benefit the safety of mining.

## 1. Introduction

Rock mechanics have always attracted the research attentions of scholars, both domestically and abroad. Swanson and Brown [1] found that the maximum stresses in all rocks tested were independent of the stress loading path. Crouch [2] believed that the compressive strength was essentially independent of the manner in which the axial and confining stresses were applied. Some in depth research revealed that the mechanical parameters of the rock mass under loading and unloading conditions were significantly different [3]. In addition, the actual excavation process included in deep engineering projects (such as the roadway excavation [4]) shares more similarities with the unloading path [5]. Compared to the loading path, the measured mechanical parameters of the unloading path tend to be more accurate [6].

Cong et al. [7] studied the effect of unloading rate on the unloading failure of marble by adopting a multi-angle macro and mesoscopic analysis method. Zheng [8] took advantage of the PFC^3D^ simulation technology and found that a greater unloading rate often led to a lower strain energy of dissipation, accompanied with faster development of rock fissures and more intense frictional slippage during failure. Li et al. [9,10] analyzed the relationship between the unloading rate and a number of variables including the release strength and rate of the stored energy in rocks and the damage based on a series of combined dynamic and static load tests. Li and his team investigated the impact of the rock damage variables on the dynamic strength, energy consumption, and the distribution of fragment, along with a rock burst tendency index corresponding to the stored stress energy. With the acoustic emission technology, Zhang et al. [11] revealed that when the unloading rate increased, the acoustic emission count rate of coal samples and the overall energy tended to decrease, while the peak count and energy peak increased.

During the unloading process, the rock was dominated by lateral expansion deformation and brittle failure, resulting in the strong expansion becoming the root cause of the sudden and severe deformation and failure [12,13]. A low unloading rate is beneficial to the degree of rock fragmentation, while a high unloading rate contributes to the brittleness of the rock mass and damage [14,15,16]. Guo, Ji, and Cao [17] also indicated that a high unloading rate may increase the rock burst possibility. With the increase of unloading rate, the maximum principal stress, maximum principal strain, minimum principal strain, and volume strain at the time of rock failure decreased along with an increasing intermediate principal strain. In addition, the starting point of capacity expansion is advanced, transforming the failure mode gradually from shear failure to tension failure which are mostly concentrated near the unloading surface [18]. Compared with the axial loading rate, the hoop unloading rate is the main reason for the change of the rock failure mode [19]. Ma et al. [20] found that the rapid unloading intensified the damage of coal samples before and after rupture, resulting in a sharp increase in the damage curve. Therefore, in actual production, the excavation speed of coal and rock mass should be controlled at a slow speed. Some scholars believed that rock masses are exposed to more damage at a moderate unloading rate than a high or a low unloading rate [21].

Hou et al. [22,23] utilized cement mortar to process roadway surrounding rock specimens, and conducted a series of excavation and unloading model tests. Some studies also indicated that the stress adjustment process of surrounding rock in roadway excavation can be simplified as the stress path of unloading confining pressure [4,5], indicating that the initial stress, unloading confining pressure rate, and loading axial pressure speed, etc., can directly affect the damage and destruction of surrounding rock. However, few relevant studies have been conducted on the mechanical tests of coal and rock mass under the aforementioned stress path conditions, its energy evolution, and practical guidance, in combination with actual engineering. The internal strain energy of the specimen still shows an increasing trend in the unloading environment [24]. However, the release rate of elastic energy was accelerated, and the damage became more violent [25]. Zhao et al. [26] found that increasing the unloading rate led to some gradual decrease in the strain energy, and under a given loading rate, more cracks were formed by consuming less energy under high unloading rate. In this study, a number of rock mechanics tests were conducted to further study the influence of various unloading rates on the mechanical properties and energy evolution of coal, aiming to provide some insights to develop the controlling strategies of surrounding rock in the excavation, construction, and management of dynamic disasters in deep mines and other deep engineering projects.

## 2. Materials and Methods

### 2.1. Test Preparation

**Figure 1:**
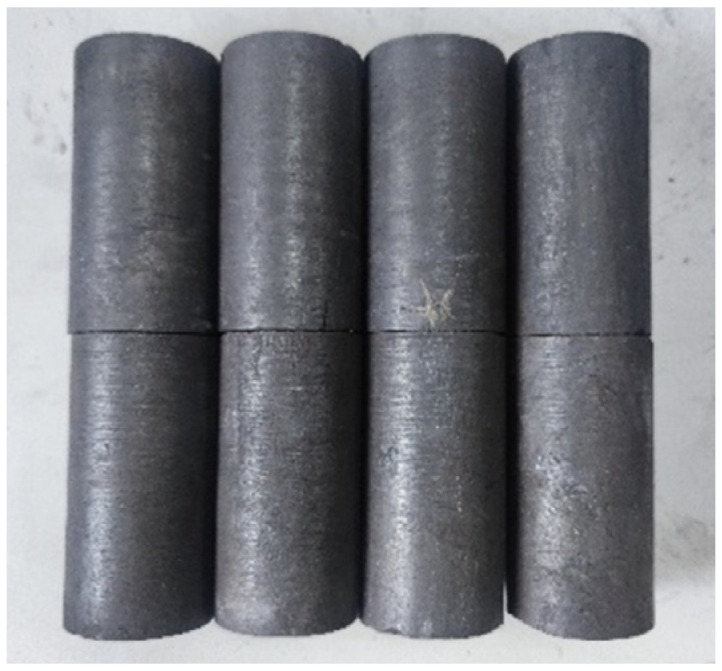
Standard test samples.

**Figure 2:**
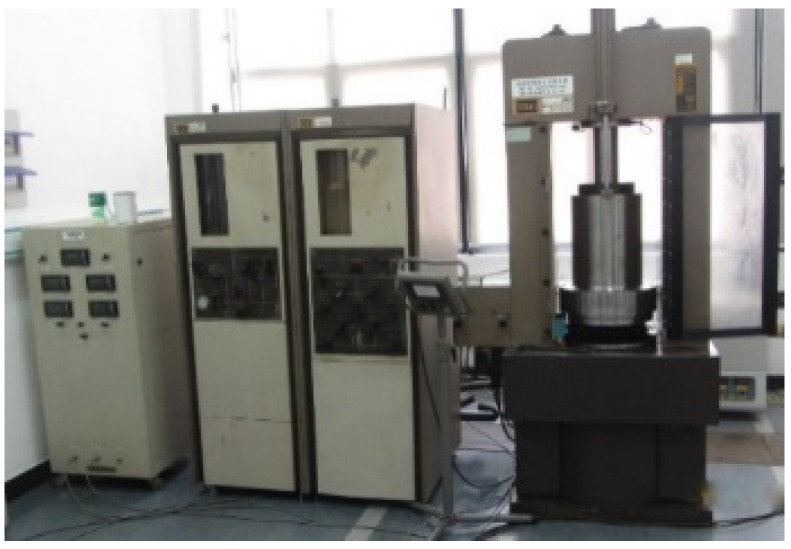
MTS815.02 rock mechanical test system.

Coal samples originated from the #8 coal seams, buried 800 m deep in the third mining area of one coal mine located in Xianyang mining zone with an average failure strength of 22.3 MPa, which were obtained by drilling with a core sleeve with a diameter of 133 mm. Following the International Rock Mechanics Test Regulation, the standard test samples were processed into a cylindrical shape with dimensions of Φ50 mm × L100 mm, as shown in Figure 1. This test was carried out via the MTS815.02 rock mechanical test system in China University of Mining and Technology, as shown in Figure 2.

### 2.2. Test Method

**Figure 3:**
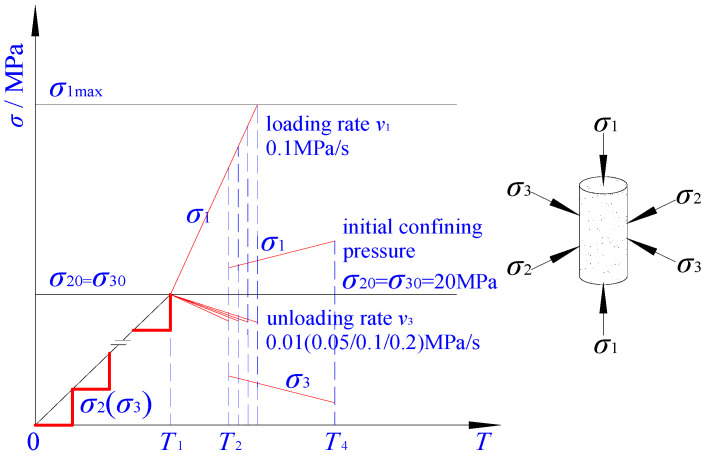
The stress path of unloading confining pressure and increasing the shaft pressure.

Based on the buried depth of the coal seam, the stress was estimated around 20 MPa in original rock status, which was selected as the original confining pressure. As demonstrated in Figure 3, the test procedure was designed and implemented to unload the confining pressure and increase the shaft pressure.

(1) Stress was controlled by increasing the load at a rate of 0.05 MPa/s, following the principle of *σ*_2_ = *σ*_3_ = 5 → *σ*_1_ = 5 → *σ*_2_ = *σ*_3_ = 10 → *σ*_1_ = 10 MPa, …. The confining pressure and shaft pressure were increased alternatively until both reached 20 MPa.
(2) In order to control the stress, the shaft pressure, *σ*_1_, was increased at a constant rate of *v*_1_ = 0.1 MPa/s; meanwhile, the confining pressure in sample *σ*_3_ was decreased at the rate of *v*_3_, following the rate setpoint of 0.01, 0.05, 0.1, and 0.2 MPa/s until the failure of the test sample_._

## 3. Result Analysis and Discussion

The deformation and failure of the test sample of coal body under various unloading rates of the confining pressure were demonstrated in Table 1.

### 3.1. The Relationship between the Unloading Rate and Mechanical Properties of the Coal Body

**Figure 4:**
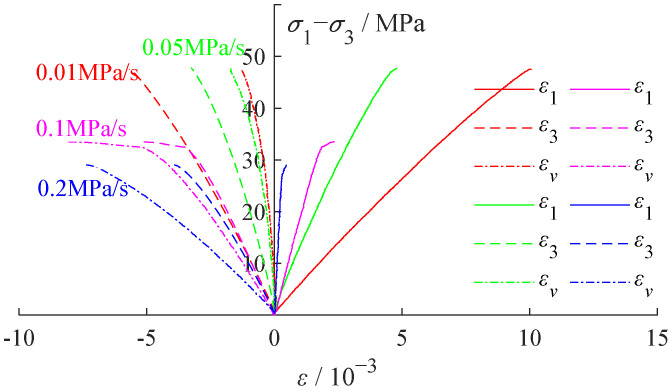
The stress–strain variance curve.

A stress–strain variance curve was developed based on the test result, as shown in Figure 4.

**Figure 5:**
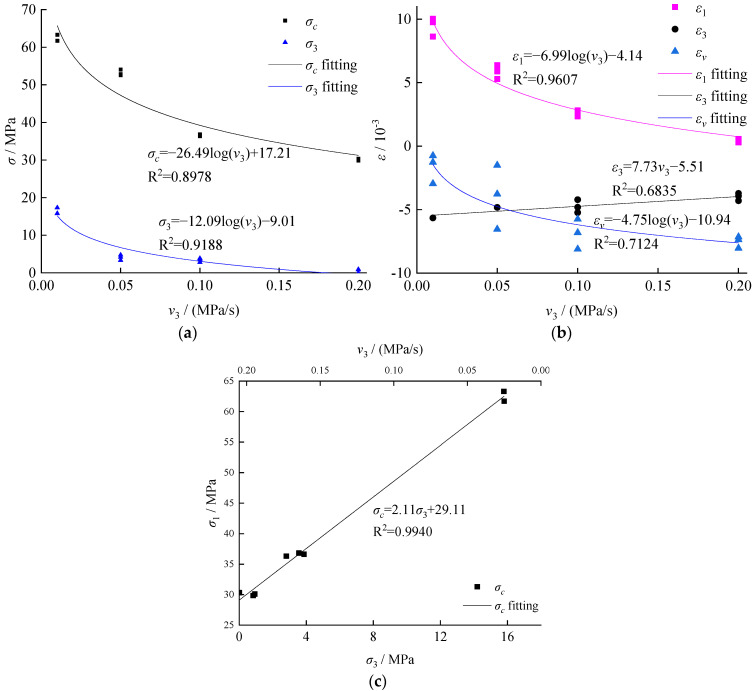
Relation among strain, stress, and unloading rate of the confining pressure: (**a**) failure strength and confining pressure, (**b**) strain, and (**c**) relation among the unloading rate of the confining pressure, the confining pressure, and failure strength.

Since the unloading point was in the state of hydrostatic pressure (original rock), the coal body was compacted. Therefore, no compaction stage was included in Figure 4. As a special engineering rock mass, the anisotropy and heterogeneity of coal were generally greater than that of general rock mass. The large discrete data was excluded for fitting analysis. As shown in Figure 5, as the unloading rate increased, the confining pressure decreased rapidly from 20 MPa, demonstrating a pattern of evolution from three-axis uniaxially, accompanied with a decreasing coal body failure strength and axial strain. Meanwhile, the volume strain increased along with the expansion, while the lateral strain demonstrated a slow decrease trend, indicating that the lateral expansion of the coal body was more sufficient under a low unloading rate, dominated by the ductility characteristics. During the failure of the coal body, the strength and confining pressure demonstrated some linear changes, indicating that a higher unloading rate tended to lead to a lower confining pressure and stress peak value during the failure, as shown in Figure 5c.

**Figure 6:**
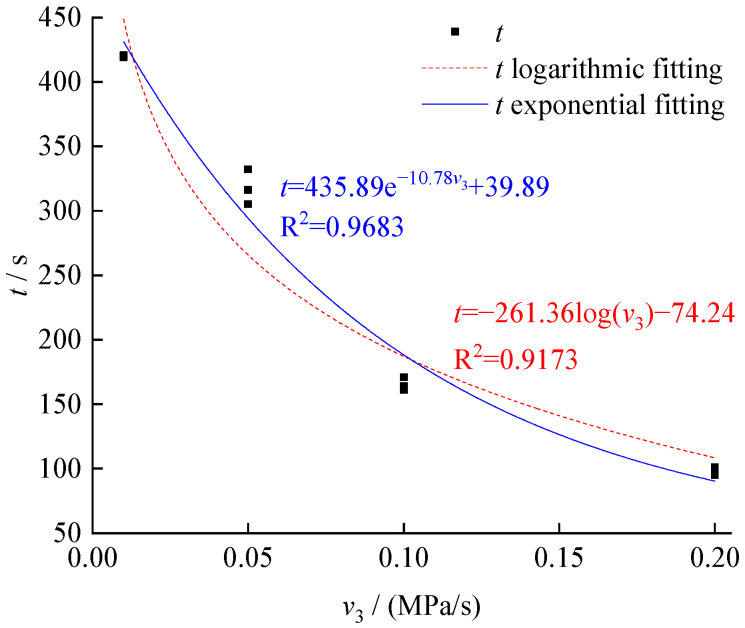
Relation between the time consumption of failure and unloading rate.

In addition, the unloading rate demonstrated a high level of impacts on the mechanical properties at the unloading rate of 0–0.1 MPa/s. When the unloading rate reached 0.2 MPa/s, the confining pressure of the failure nearly reached zero, as shown in Figure 5a, indicating that the stress environment of the coal body transformed from the three-axial compression into single axial compression. The time consumption before reaching failure under various unloading rates were presented in Figure 6, which suggests mechanical properties under an increased unloading rate and reflects the mechanical properties under the condition of single axial compression. It is worth mentioning that the fluctuations in test results were mainly caused by the test samples.

**Figure 7:**
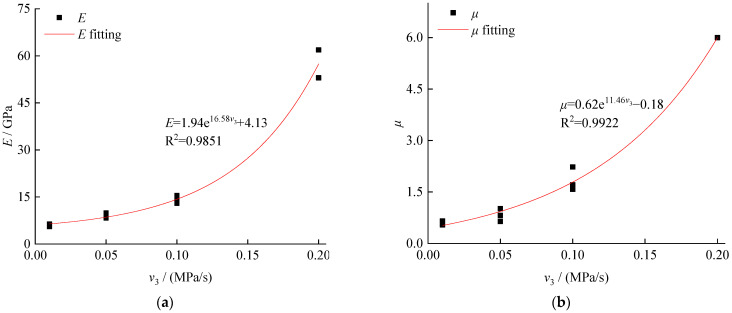
Deformation and unloading rate of the unloading rate of the confining pressure: (**a**) elastic modulus and (**b**) Poisson’s ratio.

As the coal body approached the failure, the strain and unloading rate of the confining pressure were demonstrated in Figure 7.

According to Figure 7, as the unloading rate increased, a non-linear increase was observed in the elastic modulus and Poisson’s ratio at the failure of the test samples. Considering that the Poisson’s ratio exceeded 0.5, no additional analysis was conducted. The increase in the elastic modulus contributed to the difficulties of coal body deformation, which was also reflected in the decrease of the axial strain demonstrated in Figure 5b. Meanwhile, the stronger ability to resist deformation and failure of the coal body showed more significant brittleness characteristics, compared with the condition of low unloading rate. At this time, sudden brittle failure is likely to induce some stronger dynamic disasters.

### 3.2. The Evolvement between the Unloading Rate and Energy Evolution of the Coal Body

In order to simplify the energy components in the coal body, it was assumed that the total work, *U*, of the test system during confining pressure unloading and axial pressure loading was only transformed into deformation energy, *w_e_*, and dissipation energy, *w_d_*, demonstrated as follows.

(1) $$U=U_{1}+U_{3}=w_{e}+w_{d}$$

whereas, $U_{1}=\int_{0}^{\varepsilon_{1\left(t\right)}}\sigma_{1}d\varepsilon_{1}$, $U_{3}=2\int_{0}^{\varepsilon_{3\left(t\right)}}\sigma_{3}d\varepsilon_{3}$, and $w_{e}=\frac{1}{2}\sigma_{1}\varepsilon_{1}$.

Due to the substantial energy stored in the coal body at the original rock status of 20 MPa, the average stored energy in the original rock, *w*_0_, obtained from the test was 0.0912 MJ/m^3^, resulting in a modification of Equation (1) into Equation (2).

(2) $$U=w_{0}+U_{1}+U_{3}=w_{e}+w_{d}$$

**Figure 8:**
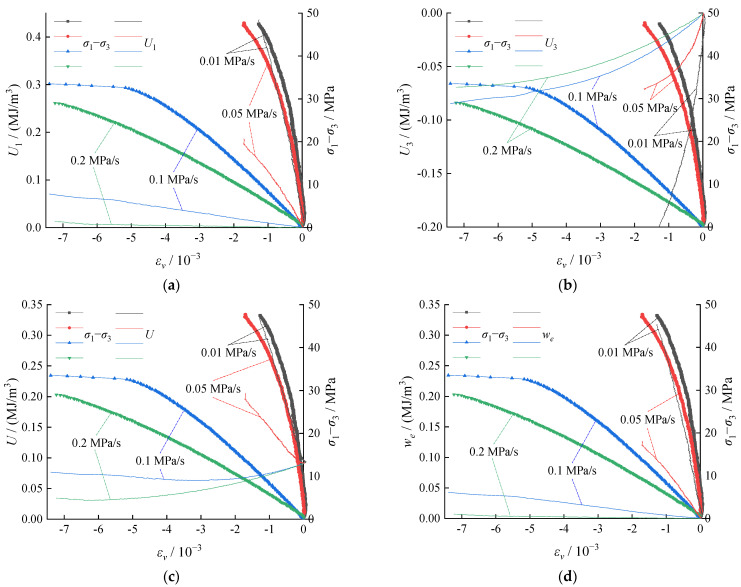

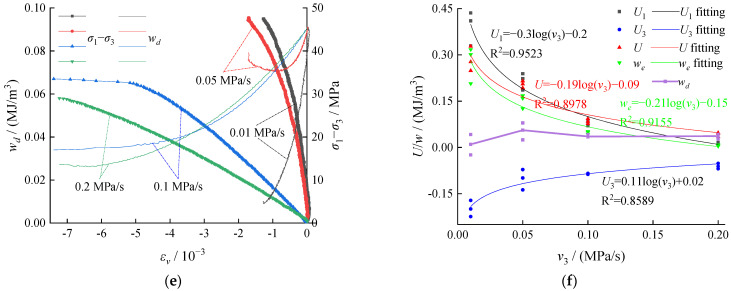
Energy evolution: (**a**) work of axial pressure, *U*_1_; (**b**) work of confining pressure, *U*_3_; (**c**) total work, *U*; (**d**) elastic strain energy, *w_e_*; (**e**) plastic strain energy, *w_d_*; and (**f**) energy and unloading rate.

Substituting the test results in Equation (2), the energy evolution in the coal body corresponding to various unloading rates of the confining pressure was demonstrated in Figure 8.

According to Figure 8a–e, as the unloading rate increased, the axial pressure of the test system did positive work, *U*_1_, while the confining pressure did negative work, *U*_3_. The total work, *U*, and releasable elastic strain energy, *w_e_*, demonstrated a decreasing trend, resulting in a less obvious law of dissipated energy, *w_d_*, for coal degradation, which is consistent with the fitting results of the test data in Figure 8f. Meanwhile, the dissipated energy, *w_d_*, for coal degradation increased in a gradually reduced rate. Under an unloading rate below 0.1 MPa/s, some erratic changes were observed in the evolutions of various types of energies. Significant differences can be observed in the same energy at the failure. When the unloading rate exceeded 0.1 MPa/s, the changes in energies gradually became limited, reaching a status of satiability. When the unloading rate exceeded a threshold point, which was similar to the energy changes under the single axial compression, the differences in strain energy observed were mainly caused by the test samples.

**Figure 9:**
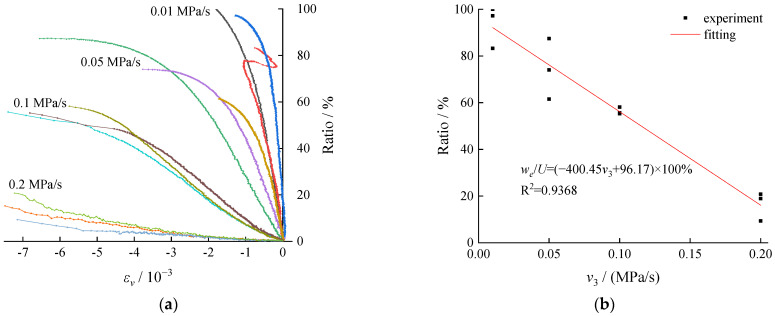
Elastic energy conversion rate: (**a**) conversion rate of elastic energy–volumetric strain evolution curve and (**b**) conversion rate of elastic strain and unloading rate.

According to research related to rock burst or impact of coal and rock mass, a higher releasable elastic strain energy stored inside can often lead to a greater risk of dynamic damage to coal and rock mass. The evolution process of elastic strain energy and the conversion rate at the time of failure of the coal specimens under different unloading rates are presented in Figure 9.

According to Figure 9a, a linear increase was observed in the conversion of the energy into the elastic strain energy during the early stage of the unloading process (elastic stage). However, more fluctuations were observed during the plastic stage. As the unloading rate increased, the elastic strain energy stored per unit volume of strain decreased, and the total energy storage of the coal body decreased at the time of failure. Figure 9b clearly shows that the elastic strain energy conversion rate decreased linearly with the increase of the unloading rate, indicating a higher unloading rate expedited the transformation from the three-axial compression to single axial compression under test conditions, and resulting in shorter time consumption to reach the failure. The input energy was reduced while the stored energy in the original rock was largely converted into dissipation energy, resulting in failure of the coal body. Despite that the failure time showed some stronger brittleness characteristics, the subsequent dynamic appearance probability and failure strength under a higher unloading speed were much lower.

### 3.3. Discussion

**Figure 10:**
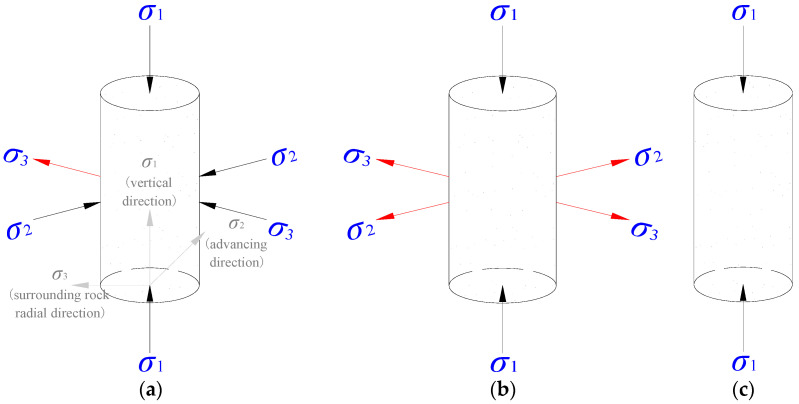
An unloading comparison between the excavation and the test: (**a**) excavation unloading, (**b**) experiment unloading, and (**c**) uniaxial compression.

Some differences were noticed between the unloading path and energy evolution observed in the test and the unloading during the actual evacuation, as demonstrated in Figure 10.

During the actual evacuation, the coal body of the study is subject to the constrain imposed by the surrounding rocks along the axial and radial direction of the roadway, resulting in unloading mainly taking place on the unrestricted side of the roadway and an unevenly distributed circular surrounding pressure, as shown in Figure 10a [27]. In the test, the unloading took place in a circular and evenly distributed pattern, resulting in an evenly distributed circular surrounding pressure, as demonstrated in Figure 10b. Under the condition of a high unloading rate, single axial loading can occur, as shown in Figure 10c, with a zero circular surrounding pressure [28]. The following conclusion can be drawn from the tests. Firstly, under the same condition, including the initial surrounding pressure, the loading rate of shaft, and the unloading rate, a lower decreasing rate of the surrounding pressure was observed in the actual excavation, demanding a higher input energy during the stress adjustment and a higher conversion rate of the releasable elastic strain energy. The coal body tends to degrade violently under a consistently high stress environment. In addition, one roadway side became less or not restricted, which increased the risks of dynamic disasters. Secondly, during the actual excavation, increasing the excavation speed was often adopted to increase the unloading rate, leading to difficulties in coal body deformation, a reduced elastic strain energy stored, and a lower conversion rate. A timely and effective support and protection system was needed to ensure safe construction, which explains the adoption of quick through excavation method under some circumstances [20]. Thirdly, regrettably, the existing research results are not yet sufficient to guide the production practice. More research on the control of the evacuation speed is needed in the future.

## 4. Conclusions

In this study, considering that the stress path of the surrounding pressure unloading and shaft pressure loading in the test can realistically reflect the stress adjustment process of the surrounding rocks during the underground excavation, a series of tests were conducted to investigate the impacts of the unloading rate on the mechanic properties of the coal body and the energy evolution law. The following conclusions are drawn based on the test results.

(1) As the unloading rate increased, a series of mechanical properties of the coal body, including the peak strength, the confining pressure, the axial strain, and horizontal strain, tended to decrease at the rupture stage while the volume strain and the elastic modulus increased, indicating that the rupture form evolved from the ductile failure to brittle failure.
(2) Regarding the energy, the axial pressure did positive work while the surrounding pressure did negative work, with decrease in the total work and the stored elastic strain energy. In addition, the dissipation energy increased, and the elastic strain energy conversion rate decreased linearly, indicating that the high unloading rate increased the possibility of dynamic disasters induced by the instantaneous brittle rupture of the coal body. Due to the low releasable elastic strain energy stored in the coal body and the low unloading rate, the strength and probability of subsequent dynamic manifestations of coal body destruction were reduced.
(3) Some differences were noticed between the actual excavation and the three axial tests. During the actual excavation, the risk and intensity of the instantaneous failure and the consequent dynamic disasters were higher. However, increasing the excavation speed in a controlled way was beneficial to the safe and efficient construction.

## Figures and Tables

**Table 1:** Test Results.

Number	Unloading Rate/(MPa/s)	Horizontal Strain *ε*_3_/10^−3^	Volume Strain *ε_v_*/10^−3^	Confining Pressure *σ*_3_/MPa	Axial Strain *ε*_1_/10^−3^	Failure Strength *σ_c_*/MPa	$\mathrm{Average}\;\mathrm{Failure}\;\mathrm{Strength}\;\bar{\sigma_{c}}/\mathrm{MPa}$
1	0.01	−6.364	−2.953	15.808	9.775	61.704	57.658
2	0.01	−4.693	−0.749	17.327	8.636	47.965	
3	0.01	−5.652	−1.271	15.793	10.033	63.306	
4	0.05	−6.458	−6.549	4.155	6.367	52.851	53.159
5	0.05	−4.830	−3.769	3.358	5.892	54.071	
6	0.05	−3.398	−1.507	4.765	5.289	52.554	
7	0.1	−5.233	−8.112	2.810	2.354	36.312	36.585
8	0.1	−4.812	−6.812	3.865	2.811	36.629	
9	0.1	−4.215	−5.750	3.561	2.681	36.814	
10	0.2	−4.302	−9.351	0.009	0.565	30.335	30.094
11	0.2	−3.722	−7.149	0.937	0.295	30.090	
12	0.2	−3.923	−7.368	0.835	0.478	29.856	

## Data Availability

The data used to support the findings of the study are available from the corresponding author upon request.
